# Variation in suspected cancer referral pathways in primary care: comparative analysis across the International Benchmarking Cancer Partnership

**DOI:** 10.3399/BJGP.2022.0110

**Published:** 2022-09-21

**Authors:** Charlotte Lynch, Samantha Harrison, Jon D Emery, Cathy Clelland, Laurence Dorman, Claire Collins, May-Lill Johansen, Ross Lawrenson, Alun Surgey, David Weller, Dorte Ejg Jarbøl, Kirubakaran Balasubramaniam, Brian D Nicholson

**Affiliations:** Policy Information and Communications, Cancer Research UK, London, UK.; Policy Information and Communications, Cancer Research UK, London, UK.; Department of General Practice and Centre for Cancer Research, University of Melbourne, Melbourne, Australia.; Primary care, British Columbia Cancer Primary Care Programme, BC Cancer, Vancouver, Canada.; Royal College of General Practitioners in Northern Ireland, Belfast, UK.; Research Unit, Irish College of General Practitioners, Dublin, Ireland.; Research Unit of General Practice, UiT The Arctic University of Norway, Tromsø, Norway.; Waikato Medical Research Centre, University of Waikato, Hamilton, New Zealand.; North Wales Centre for Primary Care Research, Bangor University, Bangor, UK.; Usher Institute, School of Clinical Sciences and Community Health, University of Edinburgh, Edinburgh, UK.; Department of Public Health, Research Unit of General Practice, University of Southern Denmark, Odense, Denmark.; Department of Public Health, Research Unit of General Practice, University of Southern Denmark, Odense, Denmark.; Nuffield Department of Primary Care Health Sciences, University of Oxford, Oxford, UK.

**Keywords:** cancer, diagnosis, primary care, referral

## Abstract

**Background:**

International variations in cancer outcomes persist and may be influenced by differences in the accessibility and organisation of cancer patient pathways. More evidence is needed to understand to what extent variations in the structure of primary care referral pathways for cancer investigation contribute to differences in the timeliness of diagnoses and cancer outcomes in different countries.

**Aim:**

To explore the variation in primary care referral pathways for the management of suspected cancer across different countries.

**Design and setting:**

Descriptive comparative analysis using mixed methods across the International Cancer Benchmarking Partnership (ICBP) countries.

**Method:**

Schematics of primary care referral pathways were developed across 10 ICBP jurisdictions. The schematics were initially developed using the Aarhus statement (a resource providing greater insight and precision into early cancer diagnosis research) and were further supplemented with expert insights through consulting leading experts in primary care and cancer, existing ICBP data, a focused review of existing evidence on the management of suspected cancer, published primary care cancer guidelines, and evaluations of referral tools and initiatives in primary care.

**Results:**

Referral pathway schematics for 10 ICBP jurisdictions were presented alongside a descriptive comparison of the organisation of primary care management of suspected cancer. Several key areas of variation across countries were identified: inflexibility of referral pathways, lack of a managed route for non-specific symptoms, primary care practitioner decision-making autonomy, direct access to investigations, and use of emergency routes.

**Conclusion:**

Analysing the differences in referral processes can prompt further research to better understand the impact of variation on the timeliness of diagnoses and cancer outcomes. Studying these schematics in local contexts may help to identify opportunities to improve care and facilitate discussions on what may constitute best referral practice.

## INTRODUCTION

International variation persists in cancer stage at diagnosis and cancer survival.^1^^–^^6^ Evidence highlights the associations between expedited diagnosis and reduced mortality, improved 1-year survival, and improved experience of care.^7^^,^^8^ These associations are not universal for all cancers, with poorer outcomes associated with shorter intervals for some cancers, and improved outcomes with longer intervals for others.^9^ Sometimes known as the J-shaped curve, this describes what happens when critically ill patients who need urgent care have some of their pathways expedited, meaning they have shorter intervals and timescales, as well as poorer outcomes.^10^ Furthermore, some countries with the highest survival rates report the longest diagnostic intervals.^2^^,^^11^ These variations suggest that there are hidden complexities, which underpin the association between time to diagnosis and cancer outcomes, which require further exploration. Primary care is a priority area for initiatives aimed at reducing diagnostic delay because most patients with symptoms of possible cancer first attend primary care.^12^^,^^13^

**Figure 1.**
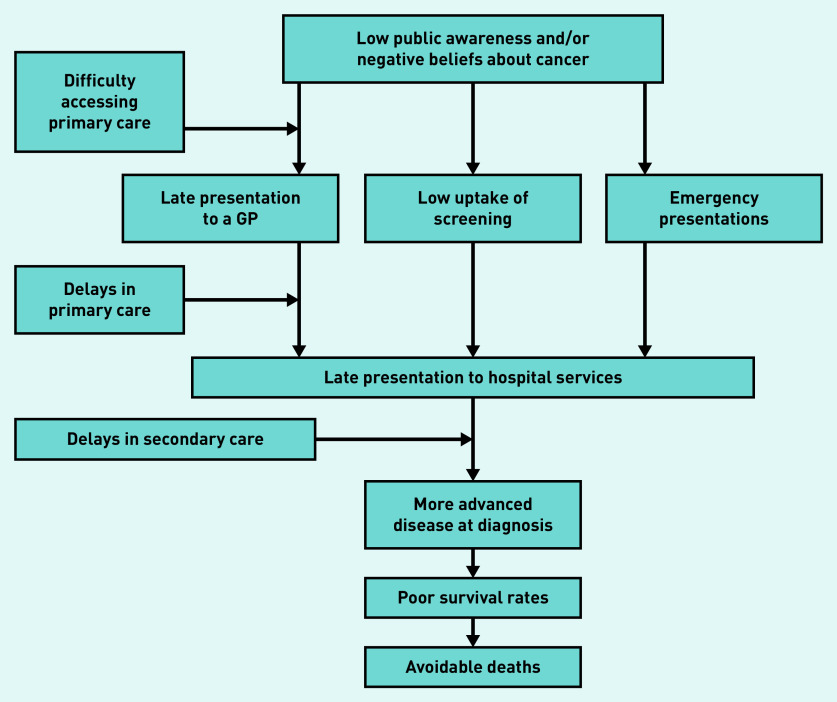
*Factors affecting primary care attendance and onward referral in suspected cancer.^18^*

Complexity is a recognised challenge in health care, which is defined by Greenhalgh and Papoutsi as *‘a dynamic and constantly emerging set of processes and objects that not only interact with each other but come to be defined by those interactions’*.^14^^–^^16^ Previous studies have used schematic and logic modelling to help visualise the complexity that exists in health care.^14^ Diagnosing cancer in primary care is complex because it is a relatively rare diagnosis and patients may present with a range of undifferentiated symptoms shared with benign illnesses.^13^^,^^17^^,^^18^ Patient-related factors (such as symptom awareness and negative beliefs about outcomes) and health system factors (such as accessibility, guidelines, capacity, and resource) influence primary care attendance and onward referral (Figure 1).^19^^–^^21^ International differences exist in primary care practitioner (PCP) responsibility for follow-up, access to investigations, and readiness to refer.^19^^,^^22^^,^^23^ Research is needed to understand whether there is international variation in the diagnostic options available to PCPs that could impact cancer outcomes, and the extent of this impact.

**Table:** How this fits in

There remains significant interest in understanding the components of primary care management of suspected cancer that may contribute to timeliness of diagnoses and cancer outcomes. This study has generated novel insights into the structure and organisation of primary care investigation of suspected cancer internationally. By comparing care between similar countries, this work can help to facilitate understanding of potential best practice in other countries and to stimulate further research to understand drivers of more favourable cancer outcomes. The schematics developed in this study can be used by clinicians to help identify opportunities and key considerations when looking to optimise referral pathways in cancer care.

The aim of this study was to map referral pathways for 10 countries in the International Cancer Benchmarking Partnership (ICBP) using pathway schematics. Identifying variations in pathways between ICBP countries may show whether the complexity of these pathways plays a role in the timeliness of cancer diagnosis and, more broadly, in variations in cancer outcomes. By generating a better understanding of the differences in primary care referral pathway, potential areas for improvement may be identified by country or jurisdiction.

## METHOD

A descriptive approach was used to develop and compare schematics of referral pathways for suspected cancer across 10 ICBP jurisdictions in 2020–2021 (Australia, British Columbia in Canada, Denmark, England, Ireland, New Zealand, Northern Ireland, Norway, Scotland, and Wales). The ICBP is a global collaboration of clinicians, policymakers, researchers, and cancer data experts, which aims to explain cancer survival differences across 21 jurisdictions in seven high-income countries with comprehensive cancer registry coverage, similar national health system expenditure, and universal access to health care. The ICBP was formed in 2009 and has consisted of jurisdictions and countries that also represent a range of cancer survival rates and have comparable key health policy issues.^24^ The selection of ICBP jurisdictions in this study was discussed and agreed with members of the ICBP Programme Board, driven by the purpose of conducting an exploratory analysis to provide an initial descriptive understanding representing the ICBP countries.

### Defining scope

**Figure 2.**
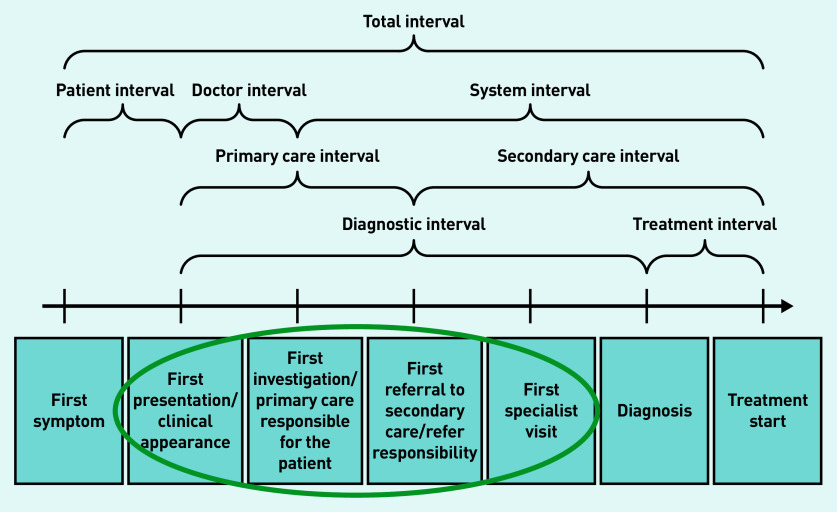
*Diagnostic steps taken from the Aarhus statement.^25^*

The study scope was informed by existing ICBP data, predominantly from studies investigating primary care referral behaviour, primary care health system mapping, and length of the cancer pathway intervals.^1^^,^^2^^,^^19^^,^^22^ Targeted searches were made of the primary care literature on the management of suspected cancer in primary care, published primary care guidelines for the investigation of suspected cancer, and evaluations of health system performance (such as audits). The findings were categorised following the diagnostic steps laid out in the Aarhus statement (a resource providing greater insight and precision into early cancer diagnosis research) (Figure 2).^25^ The following definitions were used:

- PCP assessment of cancer risk (‘First presentation/clinical appearance’);
- investigations (‘First investigation/primary care responsible for the patient’);
- onward referral (‘First referral to secondary care/refer responsibility’);
- resulting action from referral (‘First referral to secondary care/refer responsibility’ and ‘First specialist visit’ where crossover existed); and
- cancer diagnosis — this was used as an end point for the schematics, but the focus was on primary care management of suspected cancer so there is less detail on secondary care referrals and investigations leading to a confirmed diagnosis in the schematics.

The initial aim was to focus on the relative complexity of pathways, but, by supplementing the schematic development with key informant insight, it became apparent that the flexibility within the referral and investigation process for PCPs was an important differentiator between jurisdictions.

### Key informant engagement

A working group was formed of 11 leading primary care cancer research experts across the 10 ICBP countries to further develop the understanding of the international variation in the diagnostic sections highlighted in Figure 2. Each country had one representative apart from Denmark, which had two. Working group members were identified based on individual research expertise and positions held in primary care, using existing ICBP clinical networks. Semi-structured survey questions (Box 1) were developed to address evidence gaps identified during the initial scoping and targeted literature searches (see Supplementary Tables S1 and S2). Members of the working group were asked to complete the survey, which was followed up with further consultation and roundtable discussion.

**Box 1.** Semi-structured questionnaire for working group members

Please describe the referral pathways for primary care management for suspected cancer in your country. What diagnostic tests and/or investigations do primary care practitioners (PCPs) have direct access to? Are the data gathered in previous international comparisons accurate/representative, and how do they vary for different cancer sites, and across the country?^3^ What decision support tools and networks exist for PCPs to help them decide how best to investigate patients with symptoms indicative of cancer? For example, information technology tools (such as QCancer),^26^ secondary care support (such as specified nurse coordinators). At what points when investigating patients do PCPs hand over responsibility to secondary/specialist care? Are there specific processes or systems in place to avoid patients being lost from the system before a diagnosis is ruled out/confirmed (in other words, safety netting)?

### Pathway schematic development

Process flow diagrams, used to illustrate separate steps of a process in sequential order, were used to design the schematics.^27^^,^^28^ Pathway schematics were developed to reflect clinical practice up to 2019 based on the evidence identified during scoping, and the insights gained from the working group (see Supplementary Table S3). The schematics represent the diagnostic steps from initial PCP assessment for cancer risk, to PCP investigation and onward referral, then the resulting action following referral, through to cancer diagnosis (Box 2). Schematics underwent multiple rounds of review with the working group to ensure accurate reflection of primary care practice. Additional PCPs were consulted via the working group contacts where appropriate to gain wider consensus. Schematics were developed with a graphic designer using Lucid software.

**Box 2.** Schematic development: pathway step categories and definitions

**Schematic step**	**PCP assessment of cancer risk and direct access investigations**	**Referral for further investigation^a^**	**Resulting action from referral**
Pathway step overview	This step represents the initial PCP assessment of a patient in a primary care setting including simple tests PCPs can request or directly refer to (for example, bloods, urine sampling, physical assessment, and imaging) and referrals for secondary care investigations that PCPs can directly access, without requiring approval or referral from secondary care	This step details what options PCPs have for referral after patient presentation of suspected cancer. This includes designated referral pathways (where available), the existence of managed routes defined by referral guidelines, and referrals to specific healthcare settings (for example, emergency assessment or diagnostic centres). Referrals may be assigned by PCPs as urgent or standard depending on symptom presentation	This step encompasses the result of the PCP referral, likely occurring in secondary care. Predominantly, this relates to specialist assessment of the patient, including referral for investigations. This step also captures any additional steps or decision-making points after PCP referral
Pathway step definitions	**PCP assessment of cancer risk:** Initial PCP assessment of patient including: *PCP-led investigations:* Simple tests PCPs perform or refer to directly (for example, clinical examination, urine sampling, and blood tests) *PCP direct access to investigations:* PCP direct access referrals to secondary care investigations (for example, X-ray and ultrasound)	**Standard referral to specialist:** Referral from PCP to specialist under standard procedures; not along any cancer-specific or urgent referral pathways **Urgent referral to specialist:** Referral from PCP to specialist for the patient to be seen urgently; either existing as an urgent referral or via an urgent referral pathway **Referral to cancer-specific pathways:** Referral from PCP to specialist along a pathway developed specifically for patients with symptoms indicative of cancer **Access to emergency assessment:** Either PCP recommendation for the patient to attend an emergency department, or PCP requesting a specialist to see the patient on an emergency basis	**Specialist-led investigations:** Secondary care-led investigations — referral, interpretation, and follow-up — are secondary care clinicians’ responsibility **Specialist assessment of cancer risk:** Specialist examination and evaluation of patient with potential cancer, including interpretation of investigation results **Urgent access to investigations:** Expedited referral to diagnostic investigations/tests either from PCP or specialist; may be in standard hospital settings or in a specified diagnostic unit

a *Definitions for the steps in this pathway were less consistent across countries to reflect accurate practice and nuances in language used in guidance; further steps were developed during schematic development to reflect this. PCP = primary care practitioner.*

## RESULTS

The pathway schematics illustrate the steps in place to support PCP referral of suspected cancer in each jurisdiction (see Supplementary Figure S1 for details). They are organised by country according to the overall survival trends across cancer sites (1-year and 5-year survival) from the ICBP benchmarking project in descending order from highest to lowest survival.^3^ See Supplementary Table S4 for details of the processes involved at each step.

### PCP assessment of cancer risk and direct access investigations

In all jurisdictions the health systems involve initial assessment of patients in primary care, including guidance for primary care management of symptoms and test results. Direct access to simple PCP-led investigations (urine and blood tests) was universal across jurisdictions, with results being used to select patients for PCP referrals. Substantial variation existed between and within jurisdictions in the provision of direct access to specialist investigations (radiological and endoscopic), despite guidelines and recommendations supporting direct access to specialist investigation being common. It was noted that a greater ease of access by PCPs to a wide range of specialist investigations was found in Australia, particularly for radiological tests such as computed tomography scans.

### Referral for further investigation

Various mechanisms for referral exist across jurisdictions that are unmanaged (such as contacting specialists informally in Australia and Ireland) and managed (formal pathways with defined referral criteria and thresholds, for example, in Denmark and the UK). Dedicated referral pathways for vague symptoms exist in Denmark and Norway, with increasing access to these pathways in Scotland, Wales, and England. Diagnostic centres exist in various formats across jurisdictions for cancer-specific symptoms (for example, in British Columbia and Ireland) and for non-specific symptoms (for example, in Denmark, England, Norway, and Wales). Variation was noted in the route to emergency assessment, from managed routes to ensure very rapid investigation (such as in Australia), through to unmanaged routes (such as in Ireland and England). The option to refer for emergency assessment exists in Norway but is used rarely so has not been reflected in Norway’s schematic.

### Resulting action from referral

The diagnosis of cancer is confirmed by specialists in all jurisdictions, but variation exists in the organisation of specialist assessment, for example, within pathways, contacted directly, or indirectly within departments.

## DISCUSSION

### Summary

The findings of the current study add to understanding about whether health system factors contribute to international variation in cancer survival, PCP referral behaviour, and diagnostic intervals. The schematics of referral pathways for patients with suspected cancer attending primary care developed in this study illustrate high-level variation between international jurisdictions. There were notable sources of variation: PCP autonomy, flexibility of pathways, dedicated non-specific symptom pathways, and the direct access to investigations function of emergency assessment. By supplementing the schematic development with key informant insight, it became apparent that the flexibility in the referral process was a notable point of difference between jurisdictions. Autonomy refers to the ability of PCPs to investigate flexibly and refer patients they suspect may have undiagnosed cancer without referral justification or specialist triage of referrals. Referral justification aims to ensure that only the highest-risk populations of patients are investigated in systems with finite resources and can be mediated by restrictive referral guidance and criteria, and specialist triage of PCP referrals. This may become a barrier to PCP referral and investigation if clinical judgement does not align with guideline criteria or specialist opinion. Dedicated referral pathways for patients with non-specific symptoms have been/are being introduced in Denmark, Norway, England, Scotland, and Wales.^29^^–^^31^ These pathways reduce the complexity of the referral process for a group of patients who have historically fallen outside the guideline criteria. Such pathways may not be necessary in jurisdictions where there are fewer barriers to rapid investigation, as was reported to be the case in Australia.

Emerging evidence shows that patients diagnosed via emergency routes have poorer outcomes and patient experiences.^32^^,^^33^ However, it should be noted that PCPs from jurisdictions with relatively good rates of survival may access emergency assessment as a managed route to diagnosis as a solution to ensure expedited access to investigation, without being detrimental to health system resource. Further research triangulating the schematics with the proportion of emergency presentations and survival estimates will help deepen the understanding of this interaction.

### Strengths and limitations

The major strength of this study is that it provides the first international comparison of referral pathways at this level. To articulate each step from patient presentation to a PCP, through to confirmed diagnosis of cancer, is a challenging and complex task, but this study has provided a novel understanding of this landscape by using pathway schematics. They are underpinned by targeted literature searches and key informant insights from the 10 ICBP jurisdictions.

It should be acknowledged that, while the schematics are an oversimplification of clinical practice in each jurisdiction, they provide a robust baseline to understand the high-level structures and processes in place. Regional and national variations also exist in jurisdictions and between cancers. The schematics can be used to help direct future research and exploration in individual ICBP countries, and other countries outside of the ICBP, to better understand system-level drivers of more favourable survival and stage at diagnosis.

It was not possible to capture additional factors that may influence referral pathways and that could shed further light on the variation between countries in this study. These include factors such as patient demographics, socioeconomic factors, and real-world referral behaviours and practices, which can vary on a much smaller scale than was explored in this study, for example, between individual PCPs. Capturing these data comprehensively across multiple countries would be challenging and was beyond the scope for this study but should be considered for future research.

There is no established methodology for measuring complexity in cancer referral pathways, or in health care more generally. The authors developed their own approach combining targeted literature searching, guideline review, and key informant interviews. This is an area that future research could target to develop a robust and validated methodology.

### Comparison with existing literature

No comparable studies mapping cancer referral pathways were found, although there have been considerable attempts to understand health system factors influencing the timeliness of diagnosis.^1^^,^^2^^,^^14^^,^^19^^,^^22^^,^^34^^–^^36^ The role of gatekeeping, while providing greater coordination and improving access to care, has been described as a barrier to the timeliness of diagnosis, and subsequently countries have made efforts to soften this.^37^ Research has also been undertaken into the development and implementation of pathways to evaluate care, rather than exploring them from a systems approach.^38^^,^^39^ This study addresses an evidence gap, as the schematics provide an understanding of what is happening at this pathway and routes to diagnosis level, with a particular focus on primary care. It connects the understanding of international variation with snapshots of the situation in each ICBP jurisdiction, to help internal reviews to streamline pathways, supported by sharing of international practice.

### Implications for research and practice

The pathway schematics developed in this study may be used to facilitate discussions in jurisdictions about what constitutes best referral practice. Analysing the differences in referral processes may lead to initiatives to better understand the impact of variation in delays in diagnosis and to improve care in each jurisdiction. Future research should focus on understanding nuances in referral processes at a local level and between cancer site-specific pathways, by developing methodologies to map real-world referral patterns using routinely collected health system data.

Allowing more flexibility and less restrictive referral processes, with greater direct access to investigations, and open channels of communication with secondary care may lead to a timelier cancer diagnosis. Targeting interventions at these areas through policy and practice may achieve timelier diagnoses, better efficiency of care, and potentially improved outcomes. Australia consistently reports higher cancer survival and a greater proportion of people diagnosed at an early stage.^3^^–^^6^ Key informants noted flexibility in the Australian system, including direct access to investigations, PCP referral autonomy, and free movement of patients between public and private systems. However, this should be caveated with the existence of disparities in access to care, particularly for indigenous communities.^40^ In Denmark, cancer outcomes markedly improved following health system reforms, including the implementation of cancer-specific pathways, pathways for non-specific potentially serious symptoms, and coordinated diagnostic centres.^41^ These examples show that there is no ‘one size fits all’ approach, but that flexible, well-resourced, adaptable referral pathways are likely to be key components to help drive timely diagnosis.

While efforts should be made to improve the diagnostic process through better access and greater flexibility, this cannot be achieved without adequate resources, workforce, and capacity. Ensuring this should be the focus for policymakers internationally to drive improvements in care.
